# TCR engineered T cells for solid tumor immunotherapy

**DOI:** 10.1186/s40164-022-00291-0

**Published:** 2022-06-20

**Authors:** Yikai Zhang, Zhipeng Liu, Wei Wei, Yangqiu Li

**Affiliations:** 1 Guangzhou Municipality Tianhe Nuoya Bio-engineering Co. Ltd, Guangzhou, 510663 China; 2 Guangdong Cord blood bank, Guangzhou, 510663 China; 3 Department of Hematology, First Affiliated Hospital, Jinan University, Guangzhou, 510632 China; 4 Key Laboratory for Regenerative Medicine of Ministry of Education, Institute of Hematology, School of Medicine, Jinan University, 601 Huang Pu Da Dao Xi, Guangzhou, 510632 China

**Keywords:** T cell receptor, TCR-T cells, Cellular immunotherapy, Solid tumors

## Abstract

T cell immunotherapy remains an attractive approach for cancer immunotherapy. T cell immunotherapy mainly employs chimeric antigen receptor (CAR)- and T cell receptor (TCR)-engineered T cells. CAR-T cell therapy has been an essential breakthrough in treating hematological malignancies. TCR-T cells can recognize antigens expressed both on cell surfaces and in intracellular compartments. Although TCR-T cells have not been approved for clinical application, a number of clinical trials have been performed, particularly for solid tumors. In this article, we summarized current TCR-T cell advances and their potential advantages for solid tumor immunotherapy.

## Introduction

Cellular immunotherapy has shown great potential for cancer treatment. This method uses genetic engineering technology to modify T cells to endow them ability to recognize and kill tumor cells [1–4]. At present, there are two common methods for T cell immunotherapy: chimeric antigen receptor T (CAR-T) cells and T cell receptor (TCR) engineered T cells. Of these methods, CAR-T cell therapy has shown exciting results in clinical trials, and several products have been approved for the treatment of hematological malignant tumors [5–12]. Still its effects in solid tumor is unsatisfactory. Currently, TCR-T cell therapy has demonstrated encouraging potential for the treatment of solid tumors [13–16]. This review mainly summarizes the research status of anti-solid tumor immunotherapy using the tumor antigen-specific TCR-T cells.

## TCR-T cell construction

The TCR is a molecule on the surface of T cells that specifically recognizes and mediates immune responses and consists of two highly variable heterogeneous peptide chains linked by disulfide bonds. TCRs include four peptide chains, α, β, γ, and δ. α and β peptide chains form αβ TCRs, while γ and δ peptide chains form γδ TCRs [17, 18]. αβ TCRs activate the TCR signaling pathway by binding to the major histocompatibility complex (MHC) on tumor cells or antigen presenting cells (APCs), which then activates a series of intracellular proteins including CD3ζ, 70-kD zeta-associated protein (ZAP70), and nuclear factor of activated T cells 2 (NFAT2), thereby mediating T cell immune function [18, 19]. TCR-T cells are constructed by transferring a TCR gene sequence that specifically recognizes tumor antigens into T cells through genetic engineering so that the T cells have the ability to specifically kill tumor cells [1, 20]. TCR-T cells can recognize not only specific antigens on the surface of tumor cells but also intracellular antigens, which allows TCR-T cells to recognize a wider spectrum of target antigens [21, 22].

**Fig. 1:**
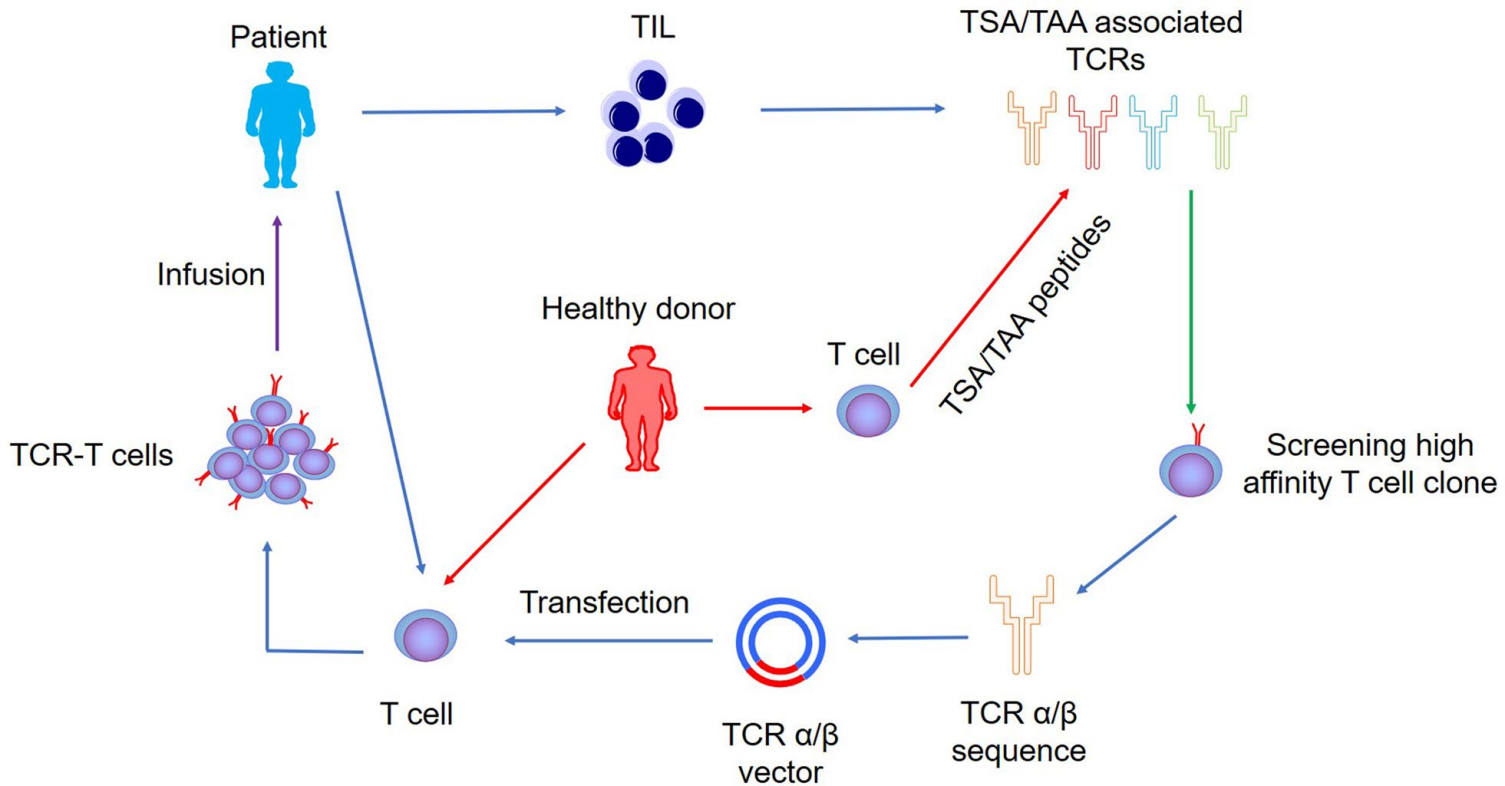
Schematic diagram of TCR-T cell construction preparation for clinical applications

To construct TCR-modified T cells, isolating and obtaining TCRs that specifically recognize tumor-specific antigen (TSA) or tumor-associated antigen (TAA) epitopes is the first step [13]. TCRs can be isolated from tumor-infiltrating T cells in the tumor tissues of patients or from healthy donor T cells induced by MHC I/II-restricted TSA or TAA peptides [23, 24]. Once a T cell clone with the highest affinity is obtained, TCR α and β chains can be cloned into the target T cell with the help of molecular cloning to form the capability to specifically recognize tumor antigens (Fig. 1) [25]. The method of obtaining tumor antigen-specific TCRs has also been improving. Our team and other researchers have found that specific TCR Vβ clones may recognize leukemia-associated antigens in leukemia patients by polymerase chain reaction (PCR) and GeneScan. We also discovered that our previously identified chronic myeloid leukemia (CML)-associated TCR Vβ clone specifically recognizes Wilm’s tumor 1 (WT1) antigenic peptides by stimulating T cells in healthy donors with MHC-restricted antigenic peptides and TCR Vβ gene sequencing [26]. Recently, our team and partners have also found TCR Vβ clones that are closely related to the prognosis of lung cancer patients [27]. Our team recently analyzed the TCR Vα and Vβ chain genes of T cells from B cell-acute lymphoid leukemia (B-ALL) patients by single-cell sequencing and found that the patients’ T cells had polyclonal subtypes, and most of them were effector subsets [28]. These results suggested that tumor antigen specifically paired TCR Vα and Vβ chain sequences can be obtained by single-cell sequencing, which can provide more accurate information for the subsequent construction of specific high-affinity TCR-T cells. Kisielow et al. proposed a new technique for screening specific T cell epitopes. Through the construction of pMHC-TCR (MCR) engineered reporter cells, this group was able to screen for MHC II T cell epitopes that specifically recognize viral and tumor antigens. The researchers also demonstrated that the tumor neoantigen-associated lymphocytes screened by this platform could protect mice from tumor cells [29]. These findings suggested that the combined application of these techniques may be able to identify and help construct individual patient tumor antigen-specific, high-affinity TCR-T cells.

### The TCR-T cell development process

Since the 1990s, a number of studies have reported that specific TCR Vα/Vβ T cell clones are found in the tumor infiltrating lymphocytes (TILs) and peripheral blood of patients with melanoma [30–34]. These clonal T cells have specific anti-melanoma cytotoxicity, and specific single-chain TCR Vβ T cells expanded in vitro have specific killing effects [33]. Fujio et al. have demonstrated that the TCRαβ gene transduction system generated by retroviruses could reconstruct the antigen-specific immunity of peripheral T cells in mice [35]. Our team also confirmed that specific TCR α/β T cells constructed by genetic engineering could effectively kill CML cells in vitro [36]. These results also suggested that both single-chain and double-chains TCR-T cells can effectively recognize and kill target cells.

Mice remain the primary model organisms for studying tumor-specific TCR-T cell therapies, and the main T cell model used is αβ^+^ CTL. In the clinical setting, dynamic analysis of the relationship between the persistence of clonal proliferative TCR Vβ subfamily T cells and disease status can provide more detailed information about specific cellular immune functions when designing immunotherapy for cancer patients. A study has shown that there was a persistent TCR Vα5 /Vβ7 CTL clone in a patient with large cell lung cancer, and this clone could still be detected 3 years after operation [37]. These data suggest that the persistence of this T cell clone may be related to the maintenance of continuous remission in this patient. Melanoma clinical trials have also shown that targeting Melan-A-specific CD8^+^ CTL cells could effectively alleviate patient conditions [34, 38]. Because γδ^+^ T cells are independent of the MHC, they also play an important role in tumor-specific immunotherapy. γδ^+^ T cells induced by donor peripheral blood could effectively kill tumor cells, e.g., Vγ9Vδ2^+^ T cells could dissolve liver cancer and rectal cancer cells but had no cytotoxic effect on normal tissues [39]. In addition, some studies have shown that a class of T cells expressing natural killer 1.1 (NK1.1) on the surface could play a cytotoxic role in tumor cells that lack expression of human leukocyte antigen-I (HLA-I) molecules. For example, the mucin 1 (MUC1) antigen is expressed in prostate cancer cells, but nearly all metastatic cancer cells lack HLA-I molecules; thus, metastatic cancer cells cannot be specifically recognized by T cells. However, this antigen may play an effective role in anti-tumor cells by stimulating NK cells [40]. Therefore γδ^+^ T cells and NK T cells were also used to construct TCR-T and TCR-NK cells [41–45].

### Preclinical study

In vitro and animal experiments of TCR-T cells were performed very early. Dembić et al. successfully transduced MHC-restricted TCRα and TCRβ genes in mouse T cells in 1986 [25]. In 1999, Clay et al. successfully transferred melanoma-specific TCR Vα and Vβ chains into human peripheral blood primary T cells and confirmed that the TCR-T cells constructed by this method could effectively kill tumor cells in vitro [46]. With the continuous progression of identification methods and experimental techniques, there are increasing studies on TCR-T cells in vitro and in animal tumor models. Abad et al. constructed a premelanosome protein 1 (PMEL-1) TCR encoding the B16 melanoma antigen gp100 in peripheral blood T cells using a retroviral vector [47], and the TCR constructed in this manner could effectively slow the development of tumor cells in B16 tumor-bearing mice. Frankel et al. demonstrated that TCR-T cells targeting tyrosinase, an enzyme involved in melanin synthesis, could also effectively kill B16/A2K(b) mouse melanoma cells in vivo [48]. This result further confirmed that the antigens recognized by TCR-T cells were not only located on the surface of cells but also in the cells. Kerkar et al. constructed MHC-I restricted melanocyte differentiation antigen100 (peml-1) TCRs in mouse CD8^+^ T cells and MHC-II restricted tyrosinase related protein 1 (TRP-1) TCR in mouse CD4^+^ T cells by a γ-retrovirus (MSGV1). This group confirmed that both MSGV1-peml-1 TCR-CD8^+^ T cells and MSGV1-TRP-1 TCR-CD4^+^ T cells could effectively kill tumor cells in B16 melanoma mice [49]. This result further clarified the function and interaction of CD8^+^ and CD4^+^ T cells during the anti-tumor process. Heemskerk et al. transduced the ovalbumin (OVA)-specific OT-1 TCRαβ gene into γδ T cells in a mouse model and confirmed that the TCR-T cells constructed in this manner could effectively kill tumor cells and avoid internal dimerization between derived TCRαβ chains [42]. These data indicate that using different types of T cells to construct TCR-T cells can maintain anti-tumor effects and avoid adverse reactions. These results also broadened the range of cell selection for the construction of TCR-T cells. Recently, Wei et al. constructed an HLA-A*02:01-restricted neoantigen library, which was transferred into HLA-matched APCs to stimulate T cells from the peripheral blood of patients. This group then screened and constructed a neoantigen-specific TCR targeting the KIAA1429D1258E mutation, and these TCR-T cells showed efficiency in killing human head and neck squamous cell carcinoma in mice [50]. This result provided a new strategy for screening mutant neoantigen-specific TCRs under conditions where it is difficult to obtain tumor tissue. Moreover, this finding indicates that the construction of TCR-T cells targeting mutant antigens and the specific killing of precise individualized immunotherapy-carrying mutant antigens may be a strategy for clinical trials.

### Clinical study

Clinical trials of TCR-T cells in cancer patients were relatively early. In 1999, Clay et al. constructed melanoma antigen recognized by T cells 1 (MART-1)-targeting TCR-T cells from human primary T cells and successfully transfused them into melanoma patients [46]. In 2006, Morgan et al. reported that they transfused TCR-T cells specifically recognizing MART-1 into 15 melanoma patients and successfully achieved remission in two patients [51]. These results further confirmed the potential of TCR-T cells that specifically recognize tumor antigens to treat tumors. The information on clinical trials of TCR-T cell therapy in solid tumors are summarized in Table 1.

**Table 1:** Major clinical trials of TCR-T cell therapies for solid tumor patients

Disease	Antigen	HLA	TCR-T cell source	Clinical trial number	Phase	Status
Melanoma	MART-1	HLA-A*02:01	PB (patient)	NCT00910650	II	Completed
	MART-1	HLA-A*02:01	PB (patient)	NCT00509288	II	Completed
	MAGE-A3	HLA-DP*04:01/04:02	PB (patient)	NCT02111850	I/II	Completed
	MAGE-C2	HLA-A*0201	PB (patient)	NCT04729543	I/II	Recruiting
	PRAME	HLA-A*02	PB (patient)	NCT03686124	I	Recruiting
	NY-ESO-1	HLA-A*0201	PB (patient)	NCT03638206	I/II	Recruiting
	MAGE-A3/12	HLA-A*02:01	PB (patient)	NCT01273181	I/II	Terminated
	NY-ESO-1	HLA-A*02:01	PB (patient)	NCT00670748	II	Terminated (A more highly selected protocol with ESO TCR opened for pts with melanoma)
	gp 100	HLA-A*02:01	PB (patient)	NCT00509496	II	Terminated (Low accrual)
	PRAME	HLA-A*02:01	PB (patient)	NCT02743611	I/II	Unknown
Lung cancer	NY-ESO-1	HLA-A*02	PB (patient)	NCT02457650	I	Unknown
	NY-ESO-1/LAGE-1a	HLA-A*02:01 HLA-A*02:05 HLA-A*02	PB (patient)	NCT03709706	Ia/IIb	Active not recruiting
Sarcoma	NY-ESO-1	HLA-A*02	PB (patient)	NCT01343043	I	Completed
	MAGE-A4^c1032^	HLA-A*02	PB (patient)	NCT03132922	I	Active not recruiting
Renal cell carcinoma	HERV-E	HLA-A*11:01	CD8^+^/CD34^+^ T cell (patient)	NCT03354390	I	Recruiting
	TRAIL	/	PB (patient)	NCT00923390	I/II	Terminated
Bladder cancer	MAGE-A10^c796^	HLA-A*02:01 HLA-A*02:06	T cells (patient)	NCT02989064	I	Completed
	MAGE-A3	HLA-A*01	PB (patient)	NCT02153905	I/II	Terminated (Slow, insufficient accrual)
Esophageal cancer	MAGE-A4	HLA-A*24:02	Lymphocytes (patient)	UMIN000002395	I	Completed
Metastatic cancer	CEA	HLA-A*02:01	PB (patient)	NCT00923806	I	Terminated (Study was terminated due to poor accrual)
Pancreatic cancer	KRAS G12D	HLA-C*08:02	TIL (patient)	NCT03935893	II	Recruiting
Hepatocellular carcinoma	HBV	HLA-A*02:01 HLA-Cw*08:01	PBMC (patient)	NCT03899415	I	Recruiting
	AFP^c332^	HLA-A*02	T cells (patient)	NCT03132792	I	Recruiting
HPV^+^ cancer	HPV-16 E6	HLA-A*02	PB (patient)	NCT02280811	I/II	Completed
	HPV-16 E7	HLA-A*02	PB (patient)	NCT02858310	I/II	Recruiting

#### Melanoma

Clinical trials of TCR-T cells in the treatment of solid tumors were first performed in patients with melanoma. A variety of melanoma-associated antigens have also been confirmed to have specific TCR therapeutic potential, and the two most attractive tumor-associated antigens were MART-1 and NY-ESO-1 [52–54].

The results of a clinical trial of targeting MART-1 TCR-T cells combined with a dendritic cell vaccine demonstrated that 9 of 13 patients (69%) had tumor regression after treatment. Among these patients, three who received non-cryopreserved cell therapy had longer persistence of MART-1 TCR-T cells, but two patients developed serious adverse acute respiratory distress events and required intervention [55]. This result suggested that fresh cells might be the ideal cell model for constructing TCR-T cells with a longer-lasting anti-tumor response, while determining how to reduce its side effects needs further investigation. Another clinical trial with targeted MART-1 TCR-T cell infusion demonstrated that 20% of 20 patients who received MART-1 TCR-T cells responded, while 19% of 16 patients who received targeted gp100 TCR-T cells responded [56]. Similarly, clinical trials also demonstrated encouraging results with 11 of 20 melanoma patients responding to targeted NY-ESO-1 TCR-T cells therapy [57]. There were also two clinical trials of targeting melanoma antigen gene A3 (MAGE-A3) TCR-T cells in the treatment of melanoma. One of them (NCT01273181) was terminated due to the varying degrees of neuronal damage, and the other one (NCT02111850) has been completed, but the results have not yet been released [58, 59]. Currently, there are a number of clinical trials of TCR-T cells for the treatment of patients with melanoma (trial registry nos. NCT02743611, NCT03686124, NCT04729543, and NCT03638206).

#### Non-small cell lung cancer

With the development of targeted drugs and immunotherapy, treatment of non-small cell lung cancer (NSCLC) has been significantly improved and has benefited many patients. Some progress has been made in TCR-T cell therapy in NSCLC, and targeting NY-ESO-1 TCR-T cells has shown potential. The result of a clinical trial demonstrated that two of four NSCLC patients who received targeted NY-ESO-1 TCR-T cell infusion had a response and no severe toxicity [60]. Another ongoing clinical trial (NCT03709706) is evaluating the safety and tolerance of TCR-T cells in advanced/recurrent NSCLC patients who were treated with NY-ESO-1/LAGE-1a TCR-T cells alone or in combination with pembrolizumab, an anti-PD-1 antibody. The result of this study contributes to a better understanding of the role of TCR-T cell therapy in NSCLC. In the clinical trial CTONG 1104, our team and partners found that TCR Vβ5–6 Jβ2 − 1, TCR Vβ20 − 1 Jβ2 − 1, and TCR Vβ24 − 1 Jβ2 − 1 in NSCLC patients with an EGFR mutation were associated with good overall survival (OS) in the gefitinib group, while TCR Vβ29 − 1 Jβ2–7 was related to good OS in the conventional chemotherapy group [27]. These results suggested that TCR-T cells, based on these specific TCRs and combined with existing targeted therapies, may benefit more NSCLC patients. Moreover, the recognition, identification, and construction of TCRs that recognize novel antigens derived from tumor mutations is also an attractive research direction.

#### Sarcoma

Because NY-ESO-1 and MAGE-A4 are expressed in approximately 80% of synovial sarcoma cells [61, 62], clinical trials of TCR-T cells directed against sarcoma mainly target these two antigens. A total of 42 patients were enrolled in a phase I/II clinical trial of NY-ESO-1 TCR-T cell therapy. The results demonstrated that one patient achieved a complete response (CR), 14 patients had a partial response (PR), 24 had stable disease (SD), and 3 had PD [63]. It was demonstrated that the expansion response of the TCR-T cells infused was related to the expression of NY-ESO-1 on patient tumor cells. Another phase I clinical trial involving NY-ESO-1 TCR-T cells in the treatment of 45 patients with advanced sarcomas reported that one patient achieved CR, 14 patients achieved PR, and 25 patients had SD [64]. In the ongoing phase I clinical trial NCT03132922, preliminary results of 16 patients with advanced synovial sarcoma targeted by MAGE-A4^c1032^ TCR-T cells demonstrated that 44% had a PR, while 50% had SD [65]. The preliminary results of these clinical trials apparently suggest that TCR-T cell immunotherapy may be a potential strategy for the treatment of sarcoma. However, more studies are needed to prove the effectiveness and safety of TCR-T cells to promote their clinical application.

#### Renal cell carcinoma

Two TCR-T cell therapy clinical trials for renal cell carcinoma (RCC) have been performed. One is a phase I/II clinical trial targeting TNF-related apoptosis-inducing ligand (TRAIL) TCR-T cells, NCT00923390, which enrolled five patients with metastatic RCC. Unfortunately, this trial was terminated 10 years after its inception, and the results were not released. Another phase I clinical trial of metastatic RCC is currently ongoing to evaluate the efficacy and safety of human endogenous retroviruses-E (HERV-E) TCR-T cells. It was reported that TCR-T cells targeting trophoblast glycoprotein (5T4) have tumor killing effects in vitro, and greater than 90% of RCC cells express the 5T4 antigen [66]. This finding indicates that 5T4 may be a potential target antigen for TCR-T cell therapy in RCC. Similarly, more in vitro and in vivo studies are needed to evaluate the efficacy and safety of 5T4-specific TCR-T cells in the treatment of RCC.

#### Bladder cancer

Some clinical trials have been conducted on TCR-T cell therapy for bladder cancer, but there were no striking results. For example, a clinical trial of MAGE-A3 HLA-A*01-restricted TCR-T cells in the treatment of metastatic bladder cancer had to be terminated because of inadequate design. In another phase I clinical trial to evaluate the efficacy of MAGE-A10 TCR-T cells in three patients with bladder cancer, the anti-tumor effects of TCR-T cells were weak despite being well tolerated [67]. In general, TCR-T cell therapy in bladder cancer needs further research.

#### Esophageal cancer

Miyahara et al. constructed TCR-T cells targeting the MAGE-A4 antigen using retroviral vectors [68]. In a clinical trial involving ten patients with esophageal cancer, MAGE-A4 TCR-T cells persisted in 5 patients for greater than 5 months, and three patients with the lowest tumor burden had an OS of greater than 27 months [69]. In this clinical trial, it was also observed that although the infused TCR-T cells persisted in the patient, there was no clinical response. This outcome may be related to the absence of lymphocyte clearance of interleukin-2 (IL-2) administration before the reinfusion of TCR-T cells. These results suggested that lymphocyte clearance pretreatment before the reinfusion of TCR-T cells may improve the efficacy of TCR-T cell therapy.

#### Colorectal cancer

Carcinoembryonic antigen (CEA) is an antigen highly expressed in metastatic colorectal cancer cells [70, 71]. In 2011, Parkhurst et al. constructed TCR-T cells targeting CEA (691–699) that proved to be effective in recognizing and targeting HLA-A*0201-restricted human CEA^+^ colon cancer cells in mouse models [72]. Although this TCR-T cell demonstrated anti-tumor capability in a clinical trial of three patients with high CEA expression, two patients had PD 5–6 months after treatment, while the other did not respond. In addition, all three patients developed severe colitis 1 week after receiving a targeted CEA TCR-T cell transfusion [72]. This type of colitis may be autoimmune colitis caused by targeting CEA TCR-T cells. These results suggest that screening antigens specifically expressed on colorectal cancer cells will be an important key step in developing the idea of using TCR-T cells for colorectal cancer immunotherapy.

#### Pancreatic cancer

More recently, Leidner et al. reported that a patient with progressive metastatic pancreatic cancer was treated with TCR-T cells and had regression of visceral metastases; the response was ongoing at 6 months (NCT03935893). These TCR-T cells were original from the autologous T cells that had been genetically engineered to clonally express two allogeneic HLA-C*08:02–restricted TCRs targeting mutant KRAS (Kirsten rat sarcoma viral oncogene homolog) G12D [73]. As we know, KRAS mutation is frequent in tumors. G12D, a single amino acid mutation of KRAS, is the most frequent mutation and is seen in diverse cancers such as pancreatic ductal adenocarcinomas [74]. The result provides the promising novel cellular immunotherapy approach for pancreatic cancer, which is the deadliest of all common cancers and lacking effective specific targeted therapy. Moreover, the KRAS G12D-TCR-T cells may be further tried to treat cancer patients who carry KRAS G12D mutation, such as NSCLC and colorectal cancer [75].

#### Hepatocellular carcinoma

Several clinical trials of TCR-T cells in hepatocellular carcinoma (HCC) are underway. These trials mainly selected NY-ESO-1 and MAGE-A1 as target antigens for TCR-T cells [76]. As TCR-T cells can recognize not only antigens on the cell surface but also intracellular antigens, alpha fetoprotein (AFP) and hepatitis B (HBV) have also become attractive target antigens for TCR-T cells in clinical trials.

The first patient who received targeted HBV TCR-T cell therapy had a transplanted liver and was hepatitis B virus surface antigen (HBsAg) negative but had an extrahepatic HCC metastasis that was HBsAg positive. The serum HBsAg level decreased significantly within 30 days after receiving an HBV TCR-T cell transfusion. Unfortunately, there was no clinical reaction, and the patient died 8 months later [77].

In another phase I clinical trial of HBV-positive HCC patients, eight patients received targeting HBV TCR-T cell (LioCyx-M) reinfusion therapy [78, 79]. In this trial, one patient had PR for 30 months, while two patients had SD, and all three patients had increased serum chemokine levels after receiving a TCR-T cell transfusion. This TCR-T cell therapy is currently in a phase II clinical trial to further evaluate its effects. These results suggest that targeting HBV TCR-T cell therapy may be effective for the treatment of HCC, but more trial results are needed to support it.

In the first ADP-A2AFP TCR-T cell clinical trial targeting HCC patients, nine were treated with TCR-T cells, and one patient achieved CR for 6 months, while six patients had SD, and two patients had PD [80]. The results of this trial suggested that targeting AFP TCR-T cells may be another effective strategy for treating HCC.

#### Human papillomavirus positive cancer

The research of TCR-T cells targeting human papillomavirus (HPV) E6 and E7 antigens in the treatment of HPV^+^ cancer has been the focus of many studies [81, 82]. At present, clinical trials have been performed on TCR-T cell therapy directed against HPV E6 and E7 antigens. These clinical trials enroll patients with head and neck tumors, cervical cancer, anal cancer, vaginal cancer, and vulvar cancer.

Doran et al. performed phase I/II clinical trials targeting HPV16 E6 TCR-T cells. Of 12 patients in this trial, two patients with anal cancer had a PR for 6 months and 3 months, respectively. One patient with vaginal cancer, one with head and neck tumors, and two with cervix uteri had SD [82]. These results indicate that targeting HPV16 E6 TCR-T cells had anti-HPV^+^ tumor cell effects. In this clinical trial, the researchers also found an IFNGR1 mutation associated with the T cell response in patients with cervical cancer. The loss of HLA-A*0201, a limiting element required by HPV16 E6 TCR-T cells, was also found in patients with PD after treatment [82]. This finding indicates that TCR-T cells may miss or fail in the treatment of HPV^+^ tumors, and further understanding of the factors and mechanisms causing these phenomena will help to improve the efficacy of HPV16 E6 TCR-T cells in the treatment of HPV^+^ tumors.

In another study targeting HPV E7 antigen, high affinity TCR-T cells with an HLA-A*020-restricted E7 (11–19) epitope was successfully constructed and proven to be effective in killing tumor cells in vitro [81]. In the clinical trial of this TCR-T cell infusion (NCT02858310), a total of 12 patients were enrolled, six patients had a PR, and four patients had SD. Among these patients, those with multiple metastasis maintained PR for 9 months after receiving TCR-T cell treatment, and most of the metastatic lesions in the body were wholly eliminated [83]. At present, phase II branch clinical trials of NCT02858310 have been opened to further evaluate the efficacy and safety of HPV E7 TCR-T cells at the maximum tolerated doses, which signals that targeted HPV E7 TCR-T cell therapy demonstrated its power in treating HPV^+^ cancer patients. We expect this therapy to benefit more patients.

## Barriers to TCR-T cell therapy

Although TCR-T cell therapy is an ideal cellular immunotherapy for cancer, it still faces obstacles that limit its application.

### TCR mismatch

TCR mismatch is an obstacle to TCR-T cell therapy. There may be some mismatching between the exogenous TCR αβ gene sequence introduced by genetically engineered T cells and the endogenous TCR αβ gene sequence of T cells [84]. The genetically engineered T cells resulting from this mismatching may recognize and attack patients’ own tissue. This phenomenon was found in a mouse model of genetically engineered T cells that were produced by a mismatched TCR, leading to graft-versus-host disease (GVHD) [85]. There are two common methods to reduce the occurrence of TCR mismatching. One is to knock out endogenous TCRs using siRNA [86, 87], zinc-finger nuclease [88], transcription activator-like effector nuclease (TALEN) [89, 90], and clustered regularly interspaced short palindromic repeats-associated protein 9 (CRISPR/Cas9) technologies [91, 92]. Another is to select γδ^+^ T cells or NK cells as a source cells to construct TCR-T cells, which can avoid the mismatching of αβTCRs to some extent [41–45].

### Nonspecific cytotoxicity

Nonspecific cytotoxicity of TCR-T cells mainly means that so-called antigen-specific TCR-T cells also attack healthy tissues expressing an antigen or epitope similar to the antigen. The teams of both Van Den Beng and Linette found fatal cardiotoxicity when they used TCR-T cells targeting MART-1 and MAGE-A3 to treat melanoma, which may be related to the high expression of MART-1 and MAGE-A3 in heart tissue [93, 94]. Parkhurst et al. also found severe colitis in three patients with metastatic colorectal cancer treated with TCR-T cells targeting CEA (691–699) [72]. Thus, the principle of selecting tumor-specific antigens as targets for constructing TCR-T cells is to avoid selecting antigens expressed in healthy tissues, particularly in important organs. Targeting neoantigens produced by tumor gene mutations may be an effective way to reduce the nonspecific cytotoxicity of TCR-T cells. In the clinical trial CTONG 1104 (the 1st generation EGFR-TKI adjuvant gefitinib improves disease-free survival (DFS) for resected EGFR-mutant NSCLC with N1/N2 metastasis), we found that significant TCR rearrangements (Vβ5-6-Jβ2 − 1, Vβ20-1-Jβ2 − 1, Vβ24-1-Jβ2 − 1, and Vβ29-1-Jβ2–7) in NSCLC patients are associated with favorable overall survival (OS) and may have a specific response to tumor gene mutations [27]. Thus, with the comprehensive application of immunome library sequencing, single-cell sequencing, and MCR engineering report cell technology, obtaining and constructing TCR-T cells targeting neoantigens may be a potential strategy for cancer immunotherapy.

### Cytokine storm

Cytokine storm is T cell immunotherapy’s most common adverse reaction [95]. Significantly elevated cytokines were detected in patients with a cytokine storm, including IL-6, interferon-γ (IFN-γ), IL-10, IL-2 receptor (IL-2R), monocyte chemotactic protein-1 (MCP-1), and macrophage inflammatory protein-1β (MIP-1β). These patients mainly had adverse reactions such as high fever, myalgia, hypotension, and dyspnea [96–98]. The severity of cytokine storms is related to tumor load [99], while reducing the tumor load before cellular immunotherapy could somewhat reduce the risk of a cytokine storm. Therefore, preventing and controlling the risk of cytokine storms can increase the safety and effectiveness of cellular immunotherapies such as TCR-T cell therapy.

### Tumor microenvironment

The tumor microenvironment (TME) is an important factor affecting T cell function. Decreased expression of chemokines, such as C-X-C motif chemokine ligand 9 (CXCL9), CXCL10, CXCL11, and intercellular cell adhesion molecule-1 (ICAM-1) adhesion molecules related to T cell infiltration in the TME can inhibit T cell infiltration to tumor sites by affecting T cell migration and adhesion [100–103]. Secondly, hypoxia in the TME could promote high expression of programmed cell death ligand 1 (PD-L1) in tumor cells, tumor-associated macrophages (TAMs), and myeloid-derived suppressor cells (MDSCs). PD-L1 binds to the programmed cell death protein 1 (PD-1) on T cells and then mediates T cell exhaustion [104]. Hypoxia can also lead to high potassium levels and an acidic environment, thus affecting the ability and activity of T cells to secrete cytokines [105]. Moreover, other immunosuppressive cells in the TME, such as regulatory T cells (Treg), MDSCs, and TAMs, can inhibit the ability of CD8^+^ T cells to recognize and kill tumor cells by secreting immunosuppressive factors such as IL-10 and transforming growth factor-β (TGF-β) [106]. Thus, the combination of targeted TME therapies may be a potential strategy for improving the efficacy of TCR-T cell immunotherapy.

## Conclusions

In summary, TCR-T cell therapy has been shown to be an attractive prospect in both in vitro studies and clinical trials for solid tumors. The advantage of such genetically engineered T cells is that they can recognize and target intracellular tumor neoantigens in solid tumors that lack specific surface tumor markers. With the development of tumor immunology and the application of new technologies, e.g., immunome library sequencing and single-cell transcriptional sequencing, TCR-T cells may have more potential for solid tumor immunotherapy.

## Data Availability

Not applicable.

## Abbreviations

AFP: Alpha fetoprotein
APCs: Antigen presenting cells
B-ALL: B cell-acute lymphoid leukemia
CAR: Chimeric antigen receptor
CEA: Carcinoembryonic antigen
CML: Chronic myeloid leukemia
CR: Complete response
CRISPR/Cas9: Clustered regularly interspaced short palindromic repeats-associated protein 9
CXCL9: C-X-C motif chemokine ligand 9
DFS: Disease-free survival
EGFR: Epidermal growth factor receptor
GVHD: Graft-versus-host disease
HBsAg: Hepatitis B virus surface antigen
HBV: Hepatitis B
HCC: Hepatocellular carcinoma
HERV-E: Human endogenous retroviruses-E
HLA-I: Human leukocyte antigen-I
HPV: Human papilloma virus
ICAM-1: Intercellular cell adhesion molecule-1
IFN-γ: Interferon-γ
IL-2: Interleukin-2
IL-2R: Interleukin-2 receptor
KRAS: Kirsten rat sarcoma viral oncogene homolog
MAGE-A3: Melanoma antigen gene A3
MART-1: Melanoma antigen recognized by T cells 1
MCR: pMHC-TCR
MCP-1: Monocyte chemotactic protein-1
MDSC: Myeloid-derived suppressor cell
MHC: Major histocompatibility complex
MIP-1β: Macrophage inflammatory protein-1β
MSGV1: γ-retrovirus
MUC1: Mucins1
NFAT2: Nuclear factor of activated T cell 2
NK 1.1: Natural killer 1.1
NSCLC: Non-small cell lung cancer
OS: Overall survival
OVA: Ovalbumin
PCR: polymerase chain reaction
PD: Progression of disease
PD-1: Programmed cell death protein 1
PD-L1: Programmed cell death ligand 1
PMEL-1: Premelanosome protein 1
PR: Partial response
RCC: Renal cell carcinoma
SD: Stable disease
TAA: Tumor-associated antigen
TAM: Tumor-associated macrophage
TALENs: Transcription activator-like effector nucleases
TCR: T cell receptor
TGF-β: Transforming growth factor-β
TILs: Tumor infiltrating lymphocytes
TME: Tumor microenvironment
TRAIL: TNF-related apoptosis-inducing ligand
Treg: Regulatory T cell
TRP-1: Tyrosinase related protein 1
TSA: Tumor-specific antigen
WT1: Wilm’s tumor 1
ZAP70: 70-kD zeta-associated protein
5T4: Trophoblast glycoprotein

## References

[1] Zhao L, Cao YJ (2019). Engineered T cell therapy for cancer in the clinic. Front Immunol.

[2] Weber EW, Maus MV, Mackall CL (2020). The emerging landscape of immune cell therapies. Cell.

[3] Ecsedi M, McAfee MS, Chapuis AG (2021). The anticancer potential of T cell receptor-engineered T cells. Trends Cancer.

[4] Zhao Q, Jiang Y, Xiang S, Kaboli PJ, Shen J, Zhao Y (2021). Engineered TCR-T cell immunotherapy in anticancer precision medicine: pros and cons. Front Immunol.

[5] Dai H, Wu Z, Jia H, Tong C, Guo Y, Ti D (2020). Bispecific CAR-T cells targeting both CD19 and CD22 for therapy of adults with relapsed or refractory B cell acute lymphoblastic leukemia. J Hematol Oncol.

[6] Huang R, Li X, He Y, Zhu W, Gao L, Liu Y (2020). Recent advances in CAR-T cell engineering. J Hematol Oncol.

[7] Tian YG, Li YL, Shao YP, Zhang Y (2020). Gene modification strategies for next-generation CAR T cells against solid cancers. J Hematol Oncol.

[8] Zhang H, Zhao P, Huang H (2020). Engineering better chimeric antigen receptor T cells. Exp Hematol Oncol.

[9] Jiang Z, Sun H, Yu J, Tian W, Song Y (2021). Targeting CD47 for cancer immunotherapy. J Hematol Oncol.

[10] Shi JZ, Zhang ZJ, Cen H, Wu H, Zhang SK, Liu JX (2021). CAR T cells targeting CD99 as an approach to eradicate T-cell acute lymphoblastic leukemia without normal blood cells toxicity. J Hematol Oncol.

[11] Su CT, Ye JC (2021). Emerging therapies for relapsed/refractory multiple myeloma: CAR-T and beyond. J Hematol Oncol.

[12] Zheng Y, Si J, Yuan T, Ding S, Tian C (2021). Immune targeted therapy for diffuse large B cell lymphoma. Blood Science.

[13] Zhang YK, Li YQ (2019). T cell receptor-engineered T cells for leukemia immunotherapy. Cancer Cell Int.

[14] Liu HT, Pan CX, Song WR, Liu DL, Li ZH, Zheng L (2021). Novel strategies for immuno-oncology breakthroughs with cell therapy. Biomark Res.

[15] Tan E, Gakhar N, Kirtane K (2021). TCR gene-engineered cell therapy for solid tumors. Best Pract Res Clin Haematol.

[16] Tsimberidou AM, Van Morris K, Vo HH, Eck S, Lin YF, Rivas JM (2021). T-cell receptor-based therapy: an innovative therapeutic approach for solid tumors. J Hematol Oncol.

[17] Zhang J, Wang L (2019). The emerging world of TCR-T cell trials against cancer: a systematic review. Technol Cancer Res Treat.

[18] Shah K, Al-Haidari A, Sun J, Kazi JU (2021). T cell receptor (TCR) signaling in health and disease. Signal Transduct Target Ther.

[19] Gao R, Zhang Y, Zeng C, Li Y (2022). The role of NFAT in the pathogenesis and targeted therapy of hematological malignancies. Eur J Pharmacol.

[20] Chandran SS, Klebanoff CA (2019). T cell receptor-based cancer immunotherapy: emerging efficacy and pathways of resistance. Immunol Rev.

[21] Chen L, Qiao DJ, Wang JT, Tian G, Wang MJ (2019). Cancer immunotherapy with lymphocytes genetically engineered with T cell receptors for solid cancers. Immunol Lett.

[22] Biernacki MA, Brault M, Bleakley M (2019). T-cell receptor-based immunotherapy for hematologic malignancies. Cancer J.

[23] Lin C, Li Y (2013). The role of peptide and DNA vaccines in myeloid leukemia immunotherapy. Cancer Cell Int.

[24] Li Y, Lin C, Schmidt CA (2012). New insights into antigen specific immunotherapy for chronic myeloid leukemia. Cancer Cell Int.

[25] Dembic Z, Haas W, Weiss S, McCubrey J, Kiefer H, von Boehmer H (1986). Transfer of specificity by murine alpha and beta T-cell receptor genes. Nature.

[26] Zhang Y, Xu L, Chen S, Zha X, Wei W, Li Y (2019). Identification of TCR Vbeta11-2-Dbeta1-Jbeta1-1 T cell clone specific for WT1 peptides using high-throughput TCRbeta gene sequencing. Biomark Res.

[27] Chen C, Liu SM, Chen Y, Ou Q, Bao H, Xu L (2022). Predictive value of TCR Vbeta-Jbeta profile for adjuvant gefitinib in EGFR mutant NSCLC from ADJUVANT-CTONG 1104 trial. JCI Insight.

[28] Wang X, Chen Y, Li Z, Huang B, Xu L, Lai J (2021). Single-Cell RNA-Seq of T Cells in B-ALL patients reveals an exhausted subset with remarkable heterogeneity. Adv Sci (Weinh).

[29] Kisielow J, Obermair F-J, Kopf M (2019). Deciphering CD4 T cell specificity using novel MHC-TCR chimeric receptors. Nat Immunol.

[30] Cole DJ, Weil DP, Shamamian P, Rivoltini L, Kawakami Y, Topalian S (1994). Identification of MART-1-specific T-cell receptors: T cells utilizing distinct T-cell receptor variable and joining regions recognize the same tumor epitope. Cancer Res.

[31] Sensi M, Traversari C, Radrizzani M, Salvi S, Maccalli C, Mortarini R (1995). Cytotoxic T-lymphocyte clones from different patients display limited T-cell-receptor variable-region gene usage in HLA-A2-restricted recognition of the melanoma antigen Melan-A/MART-1. Proc Natl Acad Sci U S A.

[32] Farina C, van der Bruggen P, Boel P, Parmiani G, Sensi M (1996). Conserved TCR usage by HLA-Cw* 1601-restricted T cell clones recognizing melanoma antigens. Int Immunol.

[33] Lake DF, Salgaller ML, van der Bruggen P, Bernstein RM, Marchalonis JJ (1999). Construction and binding analysis of recombinant single-chain TCR derived from tumor-infiltrating lymphocytes and a cytotoxic T lymphocyte clone directed against MAGE-1. Int Immunol.

[34] Jager E, Maeurer M, Hohn H, Karbach J, Jager D, Zidianakis Z (2000). Clonal expansion of Melan A-specific cytotoxic T lymphocytes in a melanoma patient responding to continued immunization with melanoma-associated peptides. Int J Cancer.

[35] Fujio K, Misaki Y, Setoguchi K, Morita S, Kawahata K, Kato I (2000). Functional reconstitution of class II MHC-restricted T cell immunity mediated by retroviral transfer of the alpha beta TCR complex. J Immunol.

[36] Zha X, Xu L, Chen S, Yang L, Zhang Y, Lu Y (2016). Generation of V alpha13/beta21 + T cell specific target CML cells by TCR gene transfer. Oncotarget.

[37] El Hage F, Vergnon I, Grunenwald D, Soria JC, Chouaib S, Mami-Chouaib F (2005). Generation of diverse mutated tumor antigen-specific cytotoxic T lymphocytes in a lung cancer patient with long survival. Oncol Rep.

[38] Mandruzzato S, Rossi E, Bernardi F, Tosello V, Macino B, Basso G (2002). Large and dissimilar repertoire of Melan-A/MART-1-specific CTL in metastatic lesions and blood of a melanoma patient. J Immunol.

[39] Bouet-Toussaint F, Cabillic F, Toutirais O, Le Gallo M, Thomas de la Pintiere C, Daniel P (2008). Vgamma9Vdelta2 T cell-mediated recognition of human solid tumors. Potential for immunotherapy of hepatocellular and colorectal carcinomas. Cancer Immunol Immunother.

[40] Wajchman HJ, Pierce CW, Varma VA, Issa MM, Petros J, Dombrowski KE (2004). Ex vivo expansion of CD8 + CD56 + and CD8 + CD56- natural killer T cells specific for MUC1 mucin. Cancer Res.

[41] van der Veken LT, Hagedoorn RS, van Loenen MM, Willemze R, Falkenburg JHF, Heemskerk MHM (2006). Alphabeta T-cell receptor engineered gammadelta T cells mediate effective antileukemic reactivity. Cancer Res.

[42] van der Veken LT, Coccoris M, Swart E, Falkenburg JHF, Schumacher TN, Heemskerk MHM (2009). alpha beta T cell receptor transfer to gamma delta T cells generates functional effector cells without mixed TCR dimers in vivo. J Immunol.

[43] Mensali N, Dillard P, Hebeisen M, Lorenz S, Theodossiou T, Myhre MR (2019). NK cells specifically TCR-dressed to kill cancer cells. Ebiomedicine.

[44] Parlar A, Sayitoglu EC, Ozkazanc D, Georgoudaki AM, Pamukcu C, Aras M (2019). Engineering antigen-specific NK cell lines against the melanoma-associated antigen tyrosinase via TCR gene transfer. Eur J Immunol.

[45] Kang S, Gao XF, Zhang L, Yang EN, Li YH, Yu L (2021). The advances and challenges of NK cell-based cancer immunotherapy. Curr Oncol.

[46] Clay TM, Custer MC, Sachs J, Hwu P, Rosenberg SA, Nishimura MI (1999). Efficient transfer of a tumor antigen-reactive TCR to human peripheral blood lymphocytes confers anti-tumor reactivity. J Immunol.

[47] Abad JD, Wrzensinski C, Overwijk W, De Witte MA, Jorritsma A, Hsu C (2008). T-cell receptor gene therapy of established tumors in a murine melanoma model. J Immunother.

[48] Frankel TL, Burns WR, Peng PD, Yu Z, Chinnasamy D, Wargo JA (2010). Both CD4 and CD8 T cells mediate equally effective in vivo tumor treatment when engineered with a highly avid TCR targeting tyrosinase. J Immunol.

[49] Kerkar SP, Sanchez-Perez L, Yang S, Borman ZA, Muranski P, Ji Y (2011). Genetic engineering of murine CD8 + and CD4 + T cells for preclinical adoptive immunotherapy studies. J Immunother.

[50] Wei T, Leisegang M, Xia M, Kiyotani K, Li N, Zeng C (2021). Generation of neoantigen-specific T cells for adoptive cell transfer for treating head and neck squamous cell carcinoma. Oncoimmunology.

[51] Morgan RA, Dudley ME, Wunderlich JR, Hughes MS, Yang JC, Sherry RM (2006). Cancer regression in patients after transfer of genetically engineered lymphocytes. Science.

[52] Lezcano C, Jungbluth AA, Nehal KS, Hollmann TJ, Busam KJ (2018). PRAME expression in melanocytic tumors. Am J Surg Pathol.

[53] Peled N, Oton AB, Hirsch FR, Bunn P (2009). MAGE A3 antigen-specific cancer immunotherapeutic. Immunotherapy.

[54] Fonteneau JF, Brilot F, Münz C, Gannagé M (2016). The tumor antigen NY-ESO-1 mediates direct recognition of melanoma cells by CD4 + T cells after intercellular antigen transfer. J Immunol.

[55] Chodon T, Comin-Anduix B, Chmielowski B, Koya RC, Wu Z, Auerbach M (2014). Adoptive transfer of MART-1 T-cell receptor transgenic lymphocytes and dendritic cell vaccination in patients with metastatic melanoma. Clin Cancer Res.

[56] Johnson LA, Morgan RA, Dudley ME, Cassard L, Yang JC, Hughes MS (2009). Gene therapy with human and mouse T-cell receptors mediates cancer regression and targets normal tissues expressing cognate antigen. Blood.

[57] Robbins PF, Kassim SH, Tran TLN, Crystal JS, Morgan RA, Feldman SA (2015). A pilot trial using lymphocytes genetically engineered with an NY-ESO-1-reactive T-cell receptor: long-term follow-up and correlates with response. Clin Cancer Res.

[58] Morgan RA, Chinnasamy N, Abate-Daga D, Gros A, Robbins PF, Zheng Z (2013). Cancer regression and neurological toxicity following anti-MAGE-A3 TCR gene therapy. J Immunother.

[59] Abate-Daga D, Hanada K-i, Davis JL, Yang JC, Rosenberg SA, Morgan RA (2013). Expression profiling of TCR-engineered T cells demonstrates overexpression of multiple inhibitory receptors in persisting lymphocytes. Blood.

[60] Xia Y, Tian X, Wang J, Qiao D, Liu X, Xiao L (2018). Treatment of metastatic non-small cell lung cancer with NY-ESO-1 specific TCR engineered-T cells in a phase I clinical trial: a case report. Oncol Lett.

[61] Iura K, Maekawa A, Kohashi K, Ishii T, Bekki H, Otsuka H (2017). Cancer-testis antigen expression in synovial sarcoma: NY-ESO-1, PRAME, MAGEA4, and MAGEA1. Hum Pathol.

[62] Thomas R, Al-Khadairi G, Roelands J, Hendrickx W, Dermime S, Bedognetti D (2018). NY-ESO-1 Based Immunotherapy of Cancer: Current Perspectives. Front Immunol.

[63] Ramachandran I, Lowther DE, Dryer-Minnerly R, Wang R, Fayngerts S, Nunez D (2019). Systemic and local immunity following adoptive transfer of NY-ESO-1 SPEAR T cells in synovial sarcoma. J Immunother Cancer.

[64] D’Angelo S, Demetri G, Van Tine B, Druta M, Glod J, Chow W (2020). Final analysis of the phase 1 trial of NY-ESO-1-specific T-cell receptor (TCR) T-cell therapy (letetresgene autoleucel; GSK3377794) in patients with advanced synovial sarcoma (SS). J Immunother Cancer.

[65] Sanderson JP, Crowley DJ, Wiedermann GE, Quinn LL, Crossland KL, Tunbridge HM (2020). Preclinical evaluation of an affinity-enhanced MAGE-A4-specific T-cell receptor for adoptive T-cell therapy. Oncoimmunology.

[66] Xu Y, Morales AJ, Cargill MJ, Towlerton AMH, Coffey DG, Warren EH (2019). Preclinical development of T-cell receptor-engineered T-cell therapy targeting the 5T4 tumor antigen on renal cell carcinoma. Cancer Immunol Immunother.

[67] Hong D, Butler M, Pachynski R, Sullivan R, Kebriaei P, Boross-Harmer S (2020). Phase I clinical trial evaluating the safety of ADP-A2M10 in patients with MAGE-A10(+) head and neck, melanoma, or urothelial tumors. J Immunother Cancer.

[68] Miyahara Y, Naota H, Wang L, Hiasa A, Goto M, Watanabe M (2005). Determination of cellularly processed HLA-A2402-restricted novel CTL epitopes derived from two cancer germ line genes, MAGE-A4 and SAGE. Clin Cancer Res.

[69] Kageyama S, Ikeda H, Miyahara Y, Imai N, Ishihara M, Saito K (2015). Adoptive transfer of MAGE-A4 T-cell receptor gene-transduced lymphocytes in patients with recurrent esophageal cancer. Clin Cancer Res.

[70] Rao H, Wu H, Huang Q, Yu Z, Zhong Z (2021). Clinical value of serum CEA, CA24-2 and CA19-9 in patients with colorectal cancer. Clin Lab..

[71] Gulhati P, Yin J, Pederson L, Schmoll H-J, Hoff P, Douillard J-Y (2020). Threshold change in CEA as a predictor of non-progression to first-line systemic therapy in metastatic colorectal cancer patients with Elevated CEA. J Natl Cancer Inst.

[72] Parkhurst MR, Yang JC, Langan RC, Dudley ME, Nathan D-AN, Feldman SA (2011). T cells targeting carcinoembryonic antigen can mediate regression of metastatic colorectal cancer but induce severe transient colitis. Mol Ther.

[73] Leidner R, Sanjuan Silva N, Huang H, Sprott D, Zheng C, Shih Y-P (2022). Neoantigen T-cell receptor gene therapy in pancreatic cancer. N Engl J Med.

[74] Melief CJM (2022). T-Cell immunotherapy against mutant KRAS for pancreatic cancer. N Engl J Med.

[75] Liu S-Y, Sun H, Zhou J-Y, Jie G-L, Xie Z, Shao Y (2020). Clinical characteristics and prognostic value of the mutation in Chinese non-small cell lung cancer patients. Biomark Res.

[76] Hendrickson PG, Olson M, Luetkens T, Weston S, Han T, Atanackovic D (2020). The promise of adoptive cellular immunotherapies in hepatocellular carcinoma. Oncoimmunology.

[77] Qasim W, Brunetto M, Gehring AJ, Xue S-A, Schurich A, Khakpoor A (2015). Immunotherapy of HCC metastases with autologous T cell receptor redirected T cells, targeting HBsAg in a liver transplant patient. J Hepatol.

[78] Meng F, Zhao J, Tan AT, Hu W, Wang S-Y, Jin J (2021). Immunotherapy of HBV-related advanced hepatocellular carcinoma with short-term HBV-specific TCR expressed T cells: results of dose escalation, phase I trial. Hepatol Int.

[79] Wang FS, Meng FP, Jin JH, Li YY, Wong RW, Tan AT (2020). Use of LioCyx-M, autologous hepatitis B virus (HBV)-Specific T cell receptor (TCR) T-cells, in advanced HBV-related hepatocellular carcinoma (HCC). J Immunother Cancer.

[80] Sangro B, Borad MJ, Hausner PF, Meyer T, Mahipal A, Goyal L (2020). Data from the third dose cohort of an ongoing study with ADP-A2AFP SPEAR T cells. J Hepatol..

[81] Jin BY, Campbell TE, Draper LM, Stevanović S, Weissbrich B, Yu Z (2018). Engineered T cells targeting E7 mediate regression of human papillomavirus cancers in a murine model. JCI Insight.

[82] Doran SL, Stevanović S, Adhikary S, Gartner JJ, Jia L, Kwong MLM (2019). T-cell receptor gene therapy for human papillomavirus-associated epithelial cancers: a first-in-human, phase I/II study. J Clin Oncol.

[83] Nagarsheth NB, Norberg SM, Sinkoe AL, Adhikary S, Meyer TJ, Lack JB (2021). TCR-engineered T cells targeting E7 for patients with metastatic HPV-associated epithelial cancers. Nat Med.

[84] van Loenen MM, de Boer R, Amir AL, Hagedoorn RS, Volbeda GL, Willemze R (2010). Mixed T cell receptor dimers harbor potentially harmful neoreactivity. Proc Natl Acad Sci U S A.

[85] Bendle GM, Linnemann C, Hooijkaas AI, Bies L, de Witte MA, Jorritsma A (2010). Lethal graft-versus-host disease in mouse models of T cell receptor gene therapy. Nat Med..

[86] Ochi T, Fujiwara H, Okamoto S, An J, Nagai K, Shirakata T (2011). Novel adoptive T-cell immunotherapy using a WT1-specific TCR vector encoding silencers for endogenous TCRs shows marked antileukemia reactivity and safety. Blood.

[87] Bunse M, Bendle GM, Linnemann C, Bies L, Schulz S, Schumacher TN (2014). RNAi-mediated TCR knockdown prevents autoimmunity in mice caused by mixed TCR dimers following TCR gene transfer. Mol Ther.

[88] Provasi E, Genovese P, Lombardo A, Magnani Z, Liu P-Q, Reik A (2012). Editing T cell specificity towards leukemia by zinc finger nucleases and lentiviral gene transfer. Nat Med.

[89] Berdien B, Mock U, Atanackovic D, Fehse B (2014). TALEN-mediated editing of endogenous T-cell receptors facilitates efficient reprogramming of T lymphocytes by lentiviral gene transfer. Gene Ther.

[90] Qasim W, Zhan H, Samarasinghe S, Adams S, Amrolia P, Stafford S (2014). Molecular remission of infant B-ALL after infusion of universal TALEN gene-edited CAR T cells. Sci Transl Med.

[91] Eyquem J, Mansilla-Soto J, Giavridis T, van der Stegen SJC, Hamieh M, Cunanan KM (2017). Targeting a CAR to the TRAC locus with CRISPR/Cas9 enhances tumour rejection. Nature.

[92] Gaj T, Gersbach CA, Barbas CF (2013). ZFN, TALEN, and CRISPR/Cas-based methods for genome engineering. Trends Biotechnol.

[93] Linette GP, Stadtmauer EA, Maus MV, Rapoport AP, Levine BL, Emery L (2013). Cardiovascular toxicity and titin cross-reactivity of affinity-enhanced T cells in myeloma and melanoma. Blood.

[94] van den Berg JH, Gomez-Eerland R, van de Wiel B, Hulshoff L, van den Broek D, Bins A (2015). Case report of a fatal serious adverse event upon administration of T cells transduced with a MART-1-specific T-cell receptor. Mol Ther.

[95] Kroschinsky F, Stölzel F, von Bonin S, Beutel G, Kochanek M, Kiehl M (2017). New drugs, new toxicities: severe side effects of modern targeted and immunotherapy of cancer and their management. Crit Care.

[96] Shatrova AN, Mityushova EV, Vassilieva IO, Aksenov ND, Zenin VV, Nikolsky NN (2016). Time-dependent regulation of IL-2R α-Chain (CD25) expression by TCR signal strength and IL-2-induced STAT5 signaling in activated human blood T lymphocytes. PLoS One.

[97] Sentman M-L, Murad JM, Cook WJ, Wu M-R, Reder J, Baumeister SH (2016). Mechanisms of acute toxicity in NKG2D chimeric antigen receptor T cell-treated mice. J Immunol.

[98] Teachey DT, Lacey SF, Shaw PA, Melenhorst JJ, Maude SL, Frey N (2016). Identification of predictive biomarkers for cytokine release syndrome after chimeric antigen receptor T-cell therapy for acute lymphoblastic leukemia. Cancer Discov.

[99] Bonifant CL, Jackson HJ, Brentjens RJ, Curran KJ (2016). Toxicity and management in CAR T-cell therapy. Mol Ther Oncolytics.

[100] Vignali D, Kallikourdis M (2017). Improving homing in T cell therapy. Cytokine Growth Factor Rev.

[101] Nagarsheth N, Wicha MS, Zou W (2017). Chemokines in the cancer microenvironment and their relevance in cancer immunotherapy. Nat Rev Immunol.

[102] Neo SY, Lundqvist A (2020). The multifaceted roles of CXCL9 within the tumor microenvironment. Adv Exp Med Biol.

[103] Bikfalvi A, Billottet C (2020). The CC and CXC chemokines: major regulators of tumor progression and the tumor microenvironment. Am J Physiol Cell Physiol.

[104] Noman MZ, Desantis G, Janji B, Hasmim M, Karray S, Dessen P (2014). PD-L1 is a novel direct target of HIF-1α, and its blockade under hypoxia enhanced MDSC-mediated T cell activation. J Exp Med.

[105] Eil R, Vodnala SK, Clever D, Klebanoff CA, Sukumar M, Pan JH (2016). Ionic immune suppression within the tumour microenvironment limits T cell effector function. Nature.

[106] Springuel L, Lonez C, Alexandre B, Van Cutsem E, Machiels J-PH, Van Den Eynde M (2019). Chimeric antigen receptor-T cells for targeting solid tumors: current challenges and existing strategies. BioDrugs.

